# Protective effect of dietary phosphorus intake on cardiovascular mortality in asthma: evidence from NHANES 1999–2018

**DOI:** 10.3389/fnut.2025.1533514

**Published:** 2025-02-25

**Authors:** Shuwen Yang, Haiyan Chen, Congyi Xie, Ning Zhang

**Affiliations:** ^1^Department of Pulmonary Medicine, Zhongshan Hospital, Fudan University (Xiamen Branch), Xiamen, China; ^2^Department of Pulmonary Medicine, Zhongshan Hospital, Fudan University, Shanghai, China; ^3^Fudan Zhangjiang Institute, Shanghai, China

**Keywords:** dietary phosphorus intake, cardiovascular mortality, asthma, NHANES, nutritional epidemiology

## Abstract

**Background:**

Asthma is associated with an increased risk of cardiovascular mortality, potentially influenced by dietary phosphorus intake through its effects on inflammation and oxidative stress.

**Methods:**

Data from 7,539 asthma patients in the National Health and Nutrition Examination Survey (NHANES) 1999–2018 cohort were analyzed using weighted Cox proportional hazards models to estimate hazard ratios (HR) and 95% confidence intervals (CI). Kaplan-Meier survival curves and a nomogram were used to assess survival probabilities and individualized risk, while restricted cubic spline (RCS) analysis evaluated non-linear dose-response relationships. Sensitivity analyses were conducted to test the robustness of the findings.

**Results:**

Higher dietary phosphorus intake was associated with reduced cardiovascular mortality (HR: 0.43; 95% CI: 0.22–0.85 for the highest vs. lowest quartile; *p* for trend = 0.018). Kaplan-Meier curves showed improved survival with increasing phosphorus intake, a result consistently supported by subgroup analyses. RCS analysis confirmed a non-linear dose-response relationship, identifying a threshold at 1,861.52 mg/day, below which higher phosphorus intake was significantly associated with lower cardiovascular mortality. However, above this threshold, the protective effect diminished. Sensitivity analyses further validated these results.

**Conclusion:**

Elevated dietary phosphorus intake is associated with reduced cardiovascular mortality in asthma patients, suggesting its potential as a dietary intervention.

## Introduction

Asthma is a chronic inflammatory disease that affects millions of people worldwide, contributing significantly to morbidity and mortality. In addition to respiratory symptoms, asthma has systemic effects, particularly its association with cardiovascular disease (CVD). This connection is believed to result from systemic inflammation, oxidative stress, and autonomic dysfunction, which collectively exacerbate vascular damage and endothelial dysfunction, thus increasing cardiovascular risk (1–3).

The health outcomes of asthma patients are influenced by various dietary and lifestyle factors. Diets high in saturated fats and low in fruits and vegetables may worsen airway inflammation, while obesity and physical inactivity contribute to heightened systemic inflammation and cardiovascular risk (4, 5). Excessive sodium intake has also been linked to oxidative stress and impaired vascular function (6, 7). These findings underscore the importance of identifying dietary components that can help mitigate these risks, especially in asthma patients who already face chronic inflammation and comorbid conditions.

Dietary phosphorus has attracted attention for its potential role in regulating vascular function and inflammation. Adequate phosphorus intake has been associated with improved cardiovascular health, including lower blood pressure, possibly through its effects on vascular smooth muscle cell function and endothelium-dependent vasodilation (8, 9). Additionally, phosphorus intake has been linked to reduced levels of inflammatory markers, such as C-reactive protein (CRP), suggesting a role in modulating systemic inflammation (10, 11).

Phosphorus may also provide indirect benefits for airway function in asthma patients. By supporting adenosine triphosphate (ATP) production, it enhances cellular energy metabolism, which could help stabilize airway smooth muscle and reduce bronchospasms during exacerbations (8, 12). Its role in maintaining acid-base balance may also improve the airway microenvironment and reduce the activation of inflammatory mediators, contributing to better respiratory health (13, 14).

Although research has explored the effects of various dietary factors on asthma and cardiovascular health, limited attention has been given to the specific role of dietary phosphorus. This study aims to evaluate the relationship between dietary phosphorus intake and cardiovascular mortality in asthma patients, addressing this important gap and providing insights that could inform dietary interventions.

## Materials and methods

### Study population

The National Health and Nutrition Examination Survey (NHANES), conducted by the Centers for Disease Control and Prevention (CDC), is a large-scale program designed to assess the health and nutritional status of the U.S. population. By integrating interviews, physical examinations, and laboratory testing, NHANES gathers comprehensive data on key health indicators, including dietary patterns, medical conditions, and environmental exposures (15–17). Utilizing a nationally representative sample, the survey provides valuable insights into health trends, identifies risk factors, and supports evidence-based public health policymaking. This study adheres to the Strengthening the Reporting of Observational Studies in Epidemiology (STROBE) guidelines, ensuring methodological rigor and ethical compliance. The study protocol was approved by the Institutional Review Board (IRB) of the National Center for Health Statistics (NCHS).

**Figure 1:**
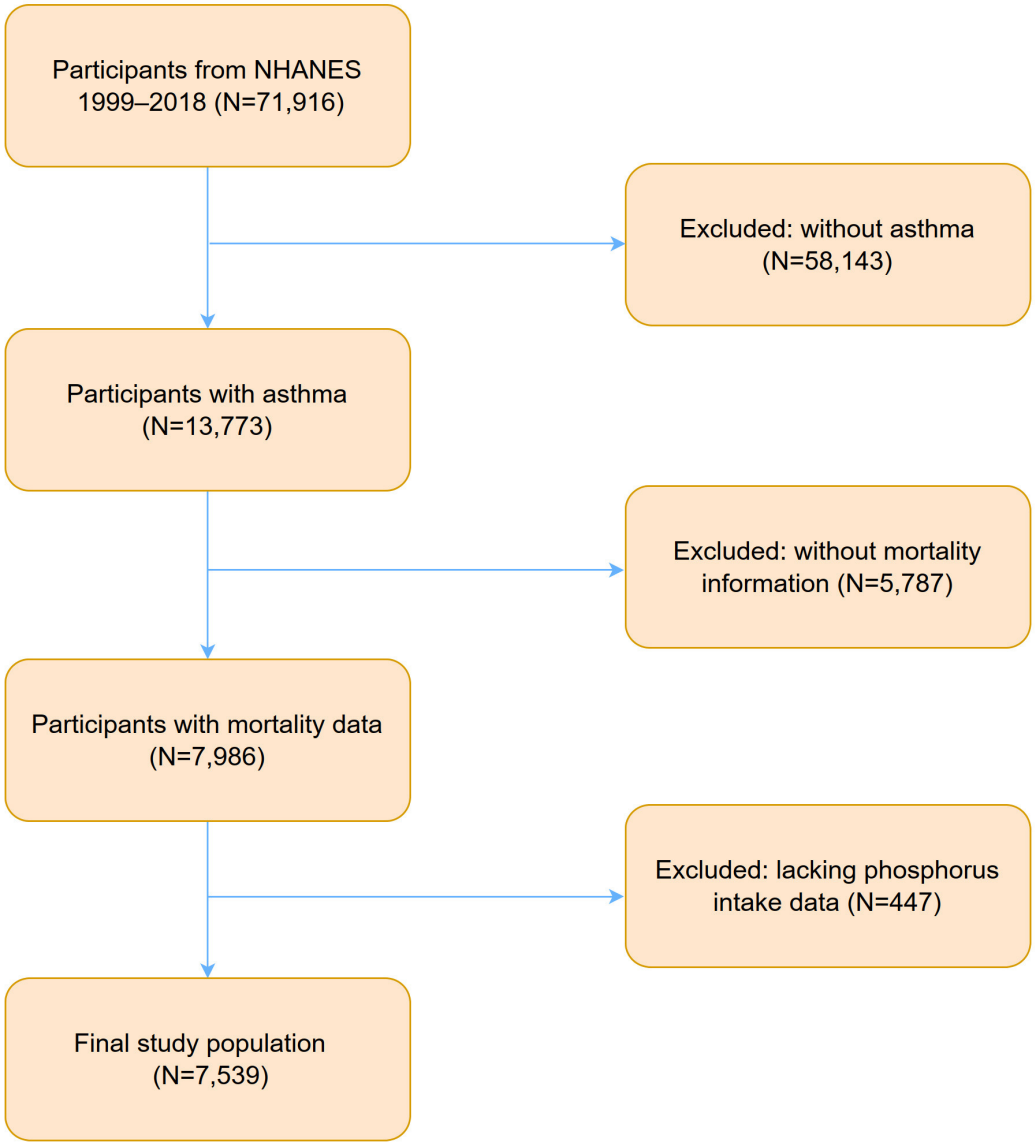
Flowchart of participants selection and exclusion from NHANES 1999–2018. NHANES, National Health and Nutrition Examination Survey.

Data for this study were derived from NHANES between 1999 and 2018, starting with an initial sample of 71,916 participants. A stepwise selection process was applied based on inclusion and exclusion criteria. First, individuals without a diagnosis of asthma were excluded (*N* = 58,143), leaving 13,773 participants identified as having asthma. Subsequently, 5,787 participants with missing mortality data were excluded, resulting in 7,986 eligible participants. An additional 447 participants lacking data of phosphorus intake were removed, reducing the sample size to 7,539. The selection process is illustrated in Figure 1.

### Definition of asthma

Asthma status was obtained from the questionnaire data in the NHANES dataset. The definition of asthma was based on the question, “Has a doctor or other health professional ever diagnosed you with asthma?” Participants who responded “Yes” were classified as having asthma. To ensure consistency in data collection, interviewers received extensive training, and the data underwent rigorous verification processes to minimize errors and biases, in line with the quality assurance standards established by NCHS. Prior research has validated the accuracy of self-reported asthma, demonstrating the reliability of this method (18, 19).

### Assessment of dietary phosphorus intake

Dietary phosphorus intake was assessed using two 24 h dietary recall interviews, based on the Food and Nutrient Database for Dietary Studies provided by the United States Department of Agriculture Available at: CDC NHANES Operations Manual. Additionally, 24 h supplement usage was recorded. The average phosphorus intake was calculated by combining dietary and supplement sources across the 2 days. Notably, data collection methods varied by survey cycle: during 1999–2002, only one 24 h dietary recall was conducted, while the 2003–2006 cycles included two recall interviews but lacked supplement usage data.

### Assessment of cardiovascular mortality

Mortality data were sourced from the 2019 Public-Use Linked Mortality Files, provided by the National Death Index (NDI) through NCHS, Division of Analysis and Epidemiology (Hyattsville, Maryland; created April 28, 2022). Following data cleaning, the variable “MORTSTAT” indicates the final mortality status, with values limited to “0” and “1,” representing presumed alive and deceased, respectively. Among deceased individuals, the variable “UCOD_LEADING” identifies the underlying leading cause of death, where a value of “001” denotes diseases of the heart as the primary cause. Other values correspond to alternative causes of death or presumed alive status. Further information is available at the CDC NCHS Mortality Data Linkage.

### Assessment of covariables

Demographic data, including age, gender, race, and body mass index (BMI), were extracted from NHANES. These data were collected through in-person interviews using validated, standardized questionnaires conducted by trained interviewers to ensure accuracy and consistency. All demographic data underwent multiple verification processes in accordance with the quality control standards established by NCHS (15). Laboratory data, including serum phosphorus and serum calcium levels, were obtained from certified laboratories. These analyses followed rigorous quality control protocols outlined by CDC, which include regular instrument calibration, the use of standard reference materials, and inter-laboratory precision testing to ensure reliability and standardization (20). Questionnaire data were used to assess smoking status, hypertension, hyperlipidemia, diabetes, chronic heart failure (CHF), coronary heart disease (CHD), renal impairment, and malignancy. These data were collected through structured, standardized interviews developed and tested by experts to ensure validity. Interviewers received extensive training to maintain consistency, and the data underwent multiple verification layers to minimize errors and biases, adhering to NCHS quality assurance guidelines (21–24). Other dietary factors, including potential confounding variables such as sodium, calcium, magnesium, total energy, and saturated fat, were assessed using two 24 h dietary recall interviews. These analyses were conducted utilizing the Food and Nutrient Database for Dietary Studies provided by the United States Department of Agriculture. Rigorous quality control procedures, including data calibration and consistency checks, were implemented to ensure the accuracy and reliability of these dietary assessments (25).

### Statistical analysis

All statistical analyses were performed using weighted methods to ensure representativeness of the NHANES cohort. Weighted analyses were conducted using the average of WTDRD1 and WTDR2D weights from the two 24 h dietary recalls in NHANES. For combined data from the 1999–2000 and 2001–2002 cycles, a four-year weight (WTMEC4YR) was applied. Sampling weights for the 1999–2018 period were calculated as follows: for 1999–2002, weights were derived as 2/10 × WTDR4YR, while for 2003–2018, weights were computed as 1/10 × the average of WTDRD1 and WTDR2D. Power calculations were not conducted a priori, as the study utilized publicly available data. The large sample size enhances statistical power by reducing variability and increasing the ability to detect meaningful effects. Additionally, the use of NHANES-provided sample weights and adjustments for the stratified multistage sampling design ensures that the estimates are nationally representative, thereby strengthening the validity of the results. This approach is consistent with previous NHANES-based studies that have produced meaningful findings without the need for a priori power analyses (26–28).

Covariates with ≤ 20% missing data were addressed using multiple imputation, while those with more than 20% missing data were excluded to ensure accurate and reliable estimates (see Supplementary Table S1). Outliers were managed using winsorization, with the 5th and 95th percentiles as cutoff thresholds. The normality of continuous variables was assessed with the Kolmogorov-Smirnov test. All continuous variables were found to be non-normally distributed and are presented as medians with interquartile ranges (IQRs). Categorical variables are reported as frequencies and percentages. Statistical comparisons were made using the Wilcoxon rank-sum test for continuous variables and the Chi-square test for categorical variables.

Cox proportional hazards regression models were used to estimate hazard ratios (HR) and 95% confidence intervals (CI). Trend tests across the three models were conducted to assess the association between dietary phosphorus intake and cardiovascular mortality in asthma patients. Model 1 adjusted for demographic factors, including age, gender, race, and BMI. Model 2 further adjusted for comorbidities such as smoking status, hypertension, hyperlipidemia, CHD, CHF, diabetes, renal impairment, and malignancy. Model 3 included additional adjustments for dietary and biochemical covariates, including sodium, calcium, magnesium, total energy, unsaturated fat, serum calcium, and serum phosphorus. Kaplan-Meier (KM) survival curves were generated to compare survival outcomes, with differences evaluated using the Log-rank test.

Subgroup analyses were performed to assess heterogeneity and interactions, stratified by age (< 60 vs. ≥60 years), gender, race (Non-Hispanic White vs. others), BMI (< 25 vs. ≥25 kg/m^2^), smoking status (ever-smokers vs. never-smokers), and the presence of comorbidities. Analyses of non-linear relationships and model performance were conducted based on Model 3. Restricted cubic spline (RCS) analysis was employed to explore non-linear relationships, while threshold effect analysis identified inflection points in the dose-mortality relationship.

Sensitivity analyses were conducted to ensure the robustness of the findings. First, phosphorus intake was examined both as a continuous variable and in quartiles, using both categorical and continuous formats. Second, models with progressively adjusted covariates (Model 1, Model 2, and Model 3) were evaluated. Third, subgroup analyses were conducted by stratifying participants based on age, gender, BMI, and the presence of comorbidities. Finally, after excluding all participants with missing data, the analyses were repeated to validate the results.

All analyses were performed using R (version 4.4.2) and Decisionlinnc software (version 1.1.1.5). A significance threshold of *p* < 0.05 was adopted, in line with its widespread use in statistical analysis as the standard for determining statistically significant results (29).

## Results

### Patient baseline and clinical characteristics

Table 1 reveals the baseline characteristics. Cardiovascular mortality was significantly associated with older age (median 68.00 years vs. median 41.00 years, *p* < 0.001) and higher median BMI (29.48 kg/m^2^ vs. 28.29 kg/m^2^, *p* = 0.039). Gender and racial distribution did not differ significantly between groups (*p* > 0.05). Smoking history was more prevalent in the cardiovascular mortality group (60.15% vs. 50.04%, *p* = 0.021). Cardiovascular mortality was also significantly associated with a higher prevalence of comorbidities, including hypertension (66.47% vs. 32.59%, *p* < 0.001), hyperlipidemia (58.28% vs. 35.21%, *p* < 0.001), diabetes (30.44% vs. 9.71%, *p* < 0.001), coronary heart disease (CHD) (18.76% vs. 3.92%, *p* < 0.001), chronic heart failure (CHF) (23.47% vs. 3.68%, *p* < 0.001), and malignancy (23.15% vs. 10.56%, *p* < 0.001). Although renal impairment was more prevalent (6.64% vs. 3.67%), the difference did not reach statistical significance (*p* = 0.039). Dietary intake in the cardiovascular mortality group was characterized by significantly lower phosphorus intake (median 1,029.80 mg vs. median 1,217.00 mg, *p* < 0.001), calcium intake (median 716.00 mg vs. median 901.00 mg, *p* < 0.001), and sodium intake (median 2,637.50 mg vs. median 3,120.00 mg, *p* < 0.001). Total energy intake (median 1,586.00 kcal vs. median 1,932.73 kcal, *p* < 0.001) and unsaturated fat intake (median 20.28 g vs. median 23.51 g, *p* < 0.001) were also significantly lower. Serum phosphorus (median 3.49 mg/dL vs. median 4.07 mg/dL, *p* < 0.001) and serum calcium (median 9.40 mg/dL vs. median 9.70 mg/dL, *p* < 0.001) levels were significantly lower in the cardiovascular mortality group.

**Table 1:** Demographic and clinical characteristics of NHANES participants.

**Characteristics**	**Overall, weighted *N =* 32,728,196**	**CVD group, weighted *N =* 840,186**	**NCVDS group, weighted *N =* 31,888,010**	***p***
Age, years	42.00 (28.00–57.00)	68.00 (55.00–78.00)	41.00 (28.00–56.00)	< 0.001
Gender				0.408
Male	13,571,805 (41.47)	377,228 (44.90)	13,194,577 (41.38)	
Female	19,156,391 (58.53)	462,957 (55.10)	18,693,434 (58.62)	
Race				0.303
Mexican American	1,794,399 (5.48)	26,462 (3.15)	1,767,937 (5.54)	
Other Hispanic	1,843,193 (5.63)	50,225 (5.98)	1,792,968 (5.62)	
Non-Hispanic White	22,495,576 (68.73)	600,498 (71.47)	21,895,079 (68.66)	
Non-Hispanic Black	4,440,551 (13.57)	135,485 (16.13)	4,305,067 (13.50)	
Other	2,154,476 (6.58)	27,517 (3.28)	2,126,960 (6.67)	
BMI, kg/m^2^	28.30 (24.10–33.85)	29.48 (25.10–36.37)	28.29 (24.10–33.75)	0.039
Phosphorus intake, mg	1,215.00 (864.00–1,629.50)	1,029.80 (703.00–1,363.50)	1,217.00 (869.50–1,634.50)	< 0.001
Calcium intake, mg	894.50 (579.50–1,331.50)	716.00 (381.00–1,216.00)	901.00 (585.50–1,332.00)	< 0.001
Sodium intake, mg	3,107.00 (2,191.00–4,190.50)	2,637.50 (1,780.00–3,570.50)	3,120.00 (2,204.50–4,201.50)	< 0.001
Total energy intake, kcal	1,925.00 (1,418.50–2,543.50)	1,586.00 (1,201.16–2,099.86)	1,932.73 (1,426.43–2,555.50)	< 0.001
Saturated fat intake, g	23.40 (15.69–33.55)	20.28 (11.63–28.85)	23.51 (15.81–33.59)	< 0.001
Serum phosphorus, mg/dL	3.80 (3.40–4.10)	3.70 (3.30–4.10)	3.80 (3.40–4.10)	0.402
Serum calcium, mg/dL	9.40 (9.20–9.60)	9.40 (9.20–9.60)	9.40 (9.20–9.60)	0.226
Smoking status				0.021
Ever	16,462,679 (50.30)	505,386 (60.15)	15,957,293 (50.04)	
Never	16,265,517 (49.70)	334,800 (39.85)	15,930,717 (49.96)	
Hypertension				< 0.001
No	21,777,907 (66.54)	281,682 (33.53)	21,496,225 (67.41)	
Yes	10,950,289 (33.46)	558,504 (66.47)	10,391,785 (32.59)	
Hyperlipidemia				< 0.001
No	21,009,584 (64.19)	350,495 (41.72)	20,659,089 (64.79)	
Yes	11,718,612 (35.81)	489,691 (58.28)	11,228,921 (35.21)	
CHD				< 0.001
No	31,319,897 (95.70)	682,550 (81.24)	30,637,347 (96.08)	
Yes	1,408,299 (4.30)	157,636 (18.76)	1,250,663 (3.92)	
CHF				< 0.001
No	31,357,617 (95.81)	642,998 (76.53)	30,714,619 (96.32)	
Yes	1,370,579 (4.19)	197,188 (23.47)	1,173,391 (3.68)	
Diabetes				< 0.001
No	29,376,434 (89.76)	584,430 (69.56)	28,792,004 (90.29)	
Yes	3,351,762 (10.24)	255,756 (30.44)	3,096,006 (9.71)	
Renal impairment				0.039
No	31,503,518 (96.26)	784,416 (93.36)	30,719,102 (96.33)	
Yes	1,224,678 (3.74)	55,770 (6.64)	1,168,908 (3.67)	
Malignancy				< 0.001
No	29,165,943 (89.12%)	645,685 (76.85%)	28,520,257 (89.44%)	
Yes	3,562,253 (10.88%)	194,500 (23.15%)	3,367,753 (10.56%)	

Continuous variables are as presented as medians with interquartile ranges (IQRs), and *P*-values were calculated using Wilcoxon rank-sum test. Categorical variables are reported as frequencies and percentages, with *P*-values determined using weighted chi-square tests.

CVD, cardiovascular death; NCVDS, non-cardiovascular death or survival; BMI, body mass index; CHD, coronary heart disease; CHF, chronic heart failure.

### Protective effect of higher dietary phosphorus intake on cardiovascular mortality

**Figure 2:**
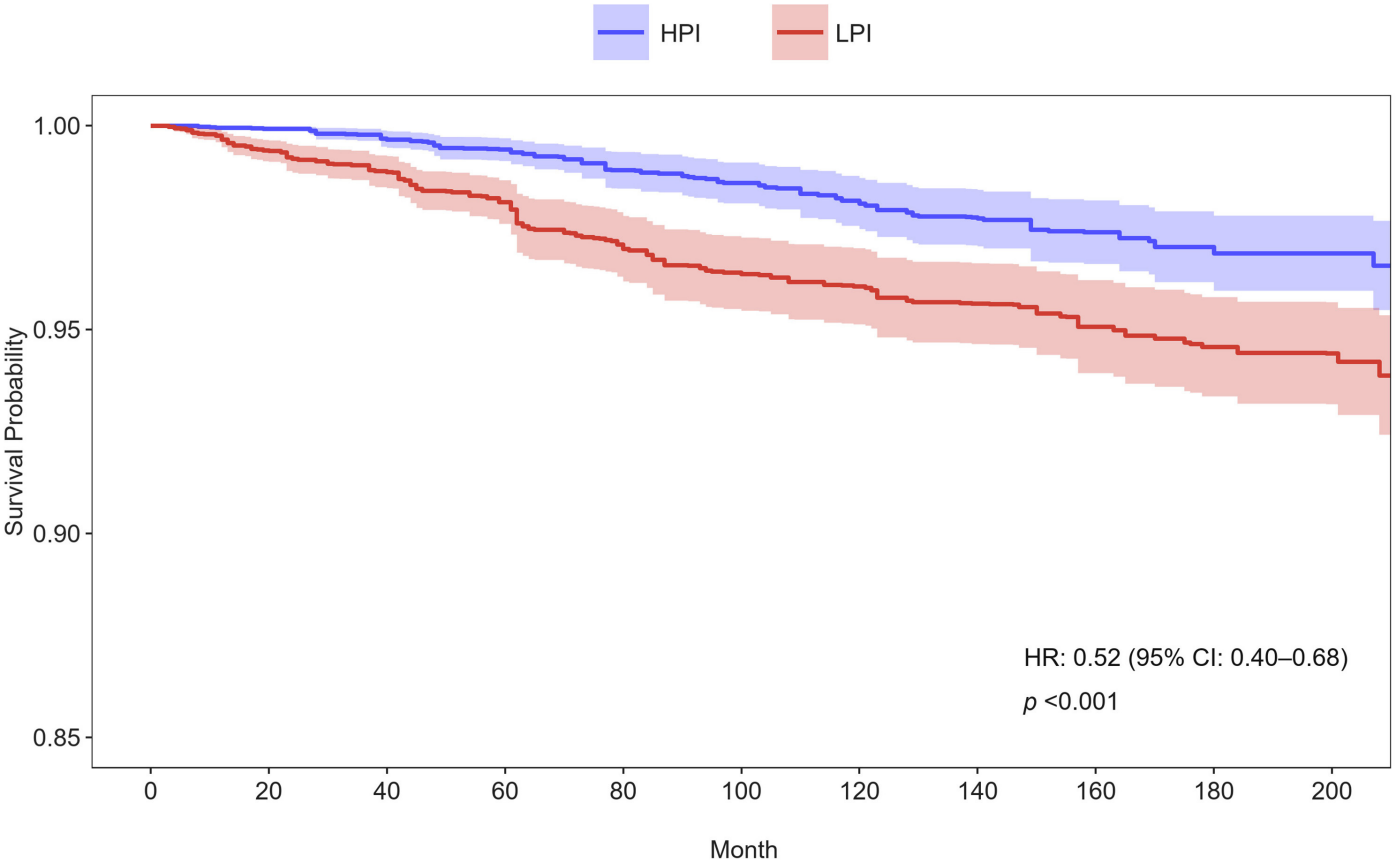
Kaplan-Meier survival curves for cardiovascular mortality stratified by phosphorus intake groups in Model 3. Participants were divided into higher and lower phosphorus intake groups based on the median intake. HPI, higher phosphorus intake; LPI, low phosphorus intake; HR, hazard ratio; CI, confidence interval.

The Cox proportional hazards regression analysis showed a significant inverse association between dietary phosphorus intake and cardiovascular mortality among asthma patients (Table 2). As a continuous variable, higher dietary phosphorus intake was consistently linked to lower cardiovascular mortality across all models (Model 1: *p* = 0.007; Model 2: *p* = 0.022; Model 3: *p* = 0.043). When analyzed by quartiles, participants in the highest quartile (Q4, ≥ 1518.69 mg/day) had significantly lower risks of cardiovascular mortality compared to those in the lowest quartile (Q1, ≤ 765.50 mg/day), with hazard ratios (HR) of 0.36 (95% CI: 0.23–0.57, *p* < 0.001) in Model 1, 0.39 (95% CI: 0.24–0.64, *p* < 0.001) in Model 2, and 0.28 (95% CI: 0.09–0.40, *p* = 0.003) in Model 3. Trend analysis across quartiles further supported a significant dose-response relationship in all models (*p* for trend < 0.001 in Model 1, < 0.001 in Model 2, and 0.013 in Model 3). Participants were categorized into higher and lower phosphorus intake groups based on the median intake, and their survival curves were compared using Kaplan-Meier analysis (Figure 2). The group with higher phosphorus intake (HPI) demonstrated significantly better survival rates compared to the lower phosphorus intake (LPI) group (*p* < 0.001), with a hazard ratio (HR) of 0.52 (95% CI: 0.40–0.68).

**Table 2:** Association between dietary phosphorus intake and cardiovascular mortality in asthma patients.

**Phosphorus intake, mg**		**Model 1**		**Model 2**		**Model 3**	
		**HR (95% CI)**	***p***	**HR (95% CI)**	***p***	**HR (95% CI)**	***p***
Continuous			0.007		0.022		0.043
Quartile1	≤ 765.50	Reference		Reference		Reference	
Quartile2	761.00–1102.50	0.59 (0.39, 0.90)	0.014	0.60 (0.39, 0.94)	0.017	0.54 (0.32, 0.93)	0.025
Quartile3	1103.00–1518.50	0.49 (0.31, 0. 76)	0.002	0.54 (0.34, 0.84)	0.007	0.44 (0.22, 0.85)	0.015
Quartile4	≥1518.69	0.36 (0.23, 0.57)	< 0.001	0.39 (0.24, 0.64)	< 0.001	0.28 (0.09, 0.40)	0.003
*p* for trend		< 0.001		< 0.001		0.013	

Model 1: Adjusted for age, gender, race, and BMI.

Model 2: Additional adjustment was made for smoking status, hypertension, hyperlipidemia, chronic heart failure, coronary heart disease, diabetes, renal impairment, and malignancy.

Model 3: Further adjustment was made for total energy intake, saturated fat intake, sodium intake, calcium intake, serum calcium, and serum phosphorus. HR, hazard ratio; CI, confidence interval.

### No interaction between subgroups

**Figure 3:**
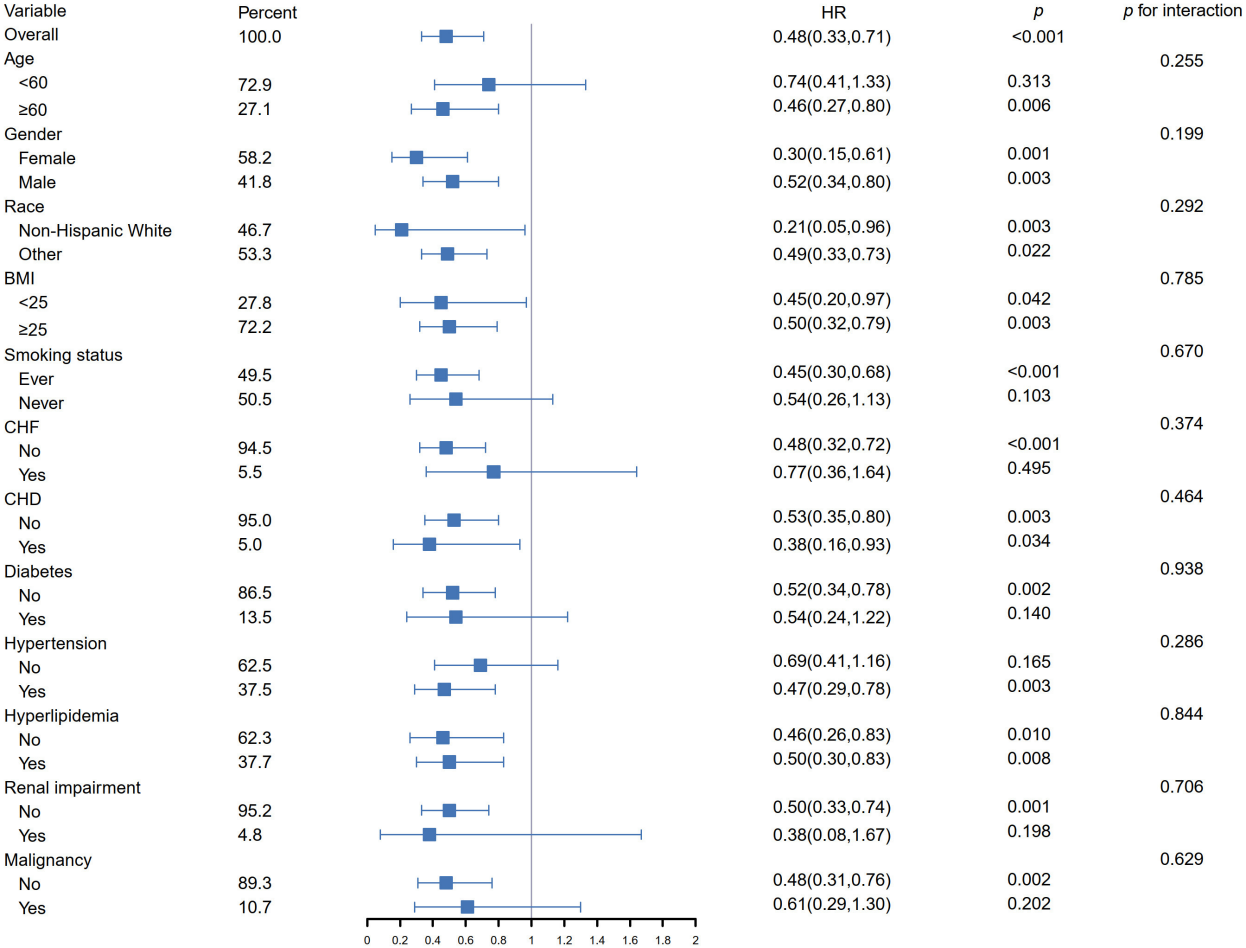
Subgroup analysis of the association between dietary phosphorus intake and cardiovascular mortality in asthma patients. HR, hazard ratio; CI, confidence interval; BMI, body mass index; CHF, chronic heart failure; CHD, coronary heart disease.

Figure 3 presents the subgroup analyses of the association between dietary phosphorus intake and cardiovascular mortality, stratified by various demographic and clinical characteristics. Higher dietary phosphorus intake was consistently associated with a protective effect across all subgroups, with hazard ratios (HR) below 1. The interaction analysis revealed no significant differences across the subgroups (all *p* for interaction >0.05), indicating that the association between phosphorus intake and cardiovascular mortality did not vary significantly based on these factors.

### Non-linear relationship with a protective effect below a threshold

**Figure 4:**
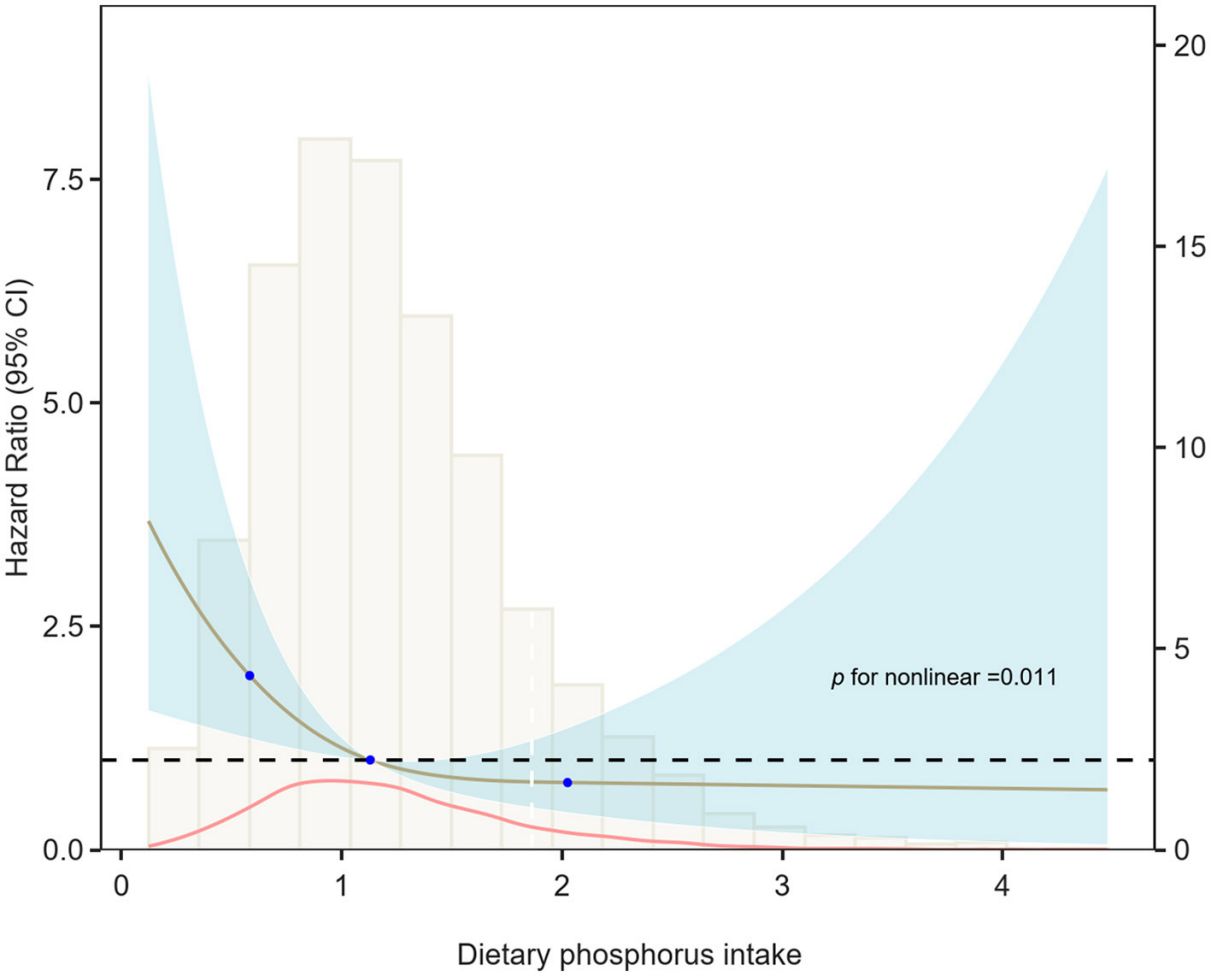
Restricted cubic spline of the relationship between dietary phosphorus intake and cardiovascular mortality risk in Model 3. The relationship between dietary phosphorus intake and cardiovascular mortality risk in asthma patients exhibited an inverted J-shaped curve, with a statistically significant non-linear association (*p* = 0.011).

The relationship between phosphorus intake and cardiovascular mortality in asthma exhibited an inverted J-shaped curve. The RCS analysis in Model 3 confirmed a nonlinear association (*p* for nonlinearity = 0.011), indicating a protective effect of phosphorus intake below a specific threshold (see Figure 4). Table 3 shows that a threshold effect was identified for phosphorus intake < 1,861.52 mg/day, where higher phosphorus intake was associated with a significantly reduced risk of cardiovascular mortality (HR: 0.39; 95% CI: 0.16–0.58). However, for phosphorus intake exceeding this threshold, although phosphorus intake appeared to be a harmful factor associated with increased cardiovascular mortality (HR: 1.74; 95% CI: 0.67–4.51), the association was not statistically significant.

**Table 3:** The threshold and saturation effect of dietary phosphorus intake on cardiovascular mortality.

	**HR (95%CI)**	***p***
Linear regression	0.55 (0.24, 0.69)	0.032
**Threshold effect**		
< 1,861.52	0.39 (0.16, 0.58)	0.024
>1,861.52	1.74 (0.67, 4.51)	0.254
Log-likelihood ratio test		0.006

A threshold effect was identified for dietary phosphorus intake below 1861.52 mg/day, showing a statistically significant association (*p* = 0.024) with a hazard ratio (HR) of 0.39 (95% CI: 0.16–0.58).

### Sensitivity analyses confirmed result consistency

Sensitivity analyses were conducted using multiple approaches. Phosphorus intake was analyzed as a continuous variable, a quartile-based categorical variable, and as a continuous variable within quartiles. The relationship between phosphorus intake and mortality was assessed using several adjustment models, including unadjusted, demographic-adjusted, and fully adjusted models. Subgroup analyses were performed by stratifying key covariates. After excluding all participants with missing data, the analyses were repeated, yielding consistent results (as detailed in Supplementary Tables S2 and Supplementary Figures S1–S3). These comprehensive analyses reinforced the robustness and reliability of the findings.

## Discussion

This study found a significant inverse association between dietary phosphorus intake and cardiovascular mortality in asthma patients, likely due to phosphorus's role in modulating inflammation and oxidative stress. Phosphorus helps reduce systemic inflammation by downregulating pro-inflammatory cytokines, such as interleukin-6 and tumor necrosis factor-α, while lowering C-reactive protein (CRP). This effect is particularly beneficial for asthma patients, where chronic inflammation contributes to endothelial dysfunction and increases cardiovascular risk (1, 2, 9). Moreover, phosphorus mitigates oxidative stress by enhancing antioxidant enzymes like superoxide dismutase and glutathione peroxidase, thereby reducing reactive oxygen species (ROS). This effect is especially important for asthma patients on long-term corticosteroid therapy, which can increase oxidative stress (8). Phosphorus metabolism is also crucial for maintaining calcium-phosphorus homeostasis, which is vital for both vascular and skeletal health. In asthma patients, chronic inflammation and corticosteroid use can disrupt this balance, potentially leading to arterial calcification and vascular damage. Adequate phosphorus intake supports stable mineral metabolism, thus reducing cardiovascular risks associated with these imbalances (30, 31). Additionally, natural phosphorus sources, such as whole grains, legumes, and dairy products, offer essential nutrients like fiber, antioxidants, and minerals that promote cardiovascular health (11, 32).

Subgroup analyses consistently demonstrated that dietary phosphorus reduces cardiovascular mortality across different age groups, genders, and comorbidities. These benefits are likely attributable to phosphorus's role in reducing inflammation, enhancing antioxidant activity, and improving endothelial function through nitric oxide synthesis and vascular relaxation (1, 2, 8–10). Furthermore, natural phosphorus sources contribute to better cholesterol metabolism, improved insulin sensitivity, and reduced vascular inflammation, which can benefit individuals with hypertension or metabolic syndrome (11, 32, 33). These findings underscore dietary phosphorus as an important modifiable factor for cardiovascular health.

Although serum phosphorus levels are associated with cardiovascular risk, dietary phosphorus intake does not directly reflect serum phosphorus concentrations. Elevated serum phosphorus is linked to vascular calcification and endothelial dysfunction, both of which contribute to cardiovascular mortality (3, 34). However, dietary phosphorus intake is influenced by the body's regulatory mechanisms and renal function, making it a better indicator of long-term exposure, particularly in non-chronic kidney disease (CKD) populations. A non-linear, inverted J-shaped relationship was observed between dietary phosphorus intake and cardiovascular mortality, with excessive phosphorus intake found to be harmful, consistent with previous studies (11, 35). Importantly, appropriate phosphorus intake may offer a protective effect against cardiovascular mortality, particularly in specific populations such as asthma patients. These results highlight the need for balanced phosphorus intake, especially in populations at risk of cardiovascular disease. Further research is required to determine optimal phosphorus intake levels for cardiovascular protection.

One limitation of this study is the lack of an a priori power analysis due to the predetermined sample size and the complex multistage design of the publicly available NHANES dataset. However, the robustness of the study was supported by several factors. The large sample size increases statistical power, reduces variability, and improves the likelihood of detecting meaningful effects (1, 2). Additionally, the use of NHANES sample weights and stratified sampling adjustments ensured nationally representative estimates, further enhancing the validity of the results (3, 4). Previous studies that have used NHANES data consistently affirm its reliability for epidemiological research (5–7).

Another limitation is that the study could not account for all relevant dietary and lifestyle factors, such as physical activity. The absence of these variables may introduce confounding effects, highlighting the need for further randomized controlled trials (RCTs) to better control for these factors. Future research should aim to provide a comprehensive evaluation of the influence of diet and lifestyle on asthma outcomes, offering more precise and actionable insights for clinical practice.

By utilizing NHANES data, this study benefits from a large, nationally representative sample, which enhances statistical power and reduces random error. The rigorous stratified multistage sampling design, combined with weighting adjustments, ensures that the findings are reflective of the U.S. population's diverse characteristics. These strengths emphasize the need for future large-scale, multicenter, and multinational RCTs to validate these results and provide vital evidence for the development of universally applicable clinical practice guidelines.

## Conclusion

Dietary phosphorus intake within an appropriate range is associated with a protective effect against cardiovascular mortality in asthma patients. Future randomized trials and mechanistic studies are needed to confirm these findings and elucidate the underlying pathways.

## Data Availability

The datasets presented in this study can be found in online repositories. The names of the repository/repositories and accession number(s) can be found below: https://wwwn.cdc.gov/nchs/nhanes/Default.aspx.
