# Supplementary material for: Protective effect of dietary phosphorus intake on cardiovascular mortality in asthma: evidence from NHANES 1999–2018
Source: Front Nutr. 2025 Feb 25;12:1533514. doi: 10.3389/fnut.2025.1533514 (PMC11893399; doi:10.3389/fnut.2025.1533514)
Supplement: Supplementary file 1 [file Data_Sheet_1.docx]

| Variable Names (N=7,539) | Missing |
| --- | --- |
| Age | 0% |
| BMI | 1.5% |
| Gender | 0% |
| Race | 0% |
| Serum phosphorus | 6.3% |
| Serum calcium | 6.4% |
| Total energy intake | 0% |
| Saturated fat intake | 7.3% |
| Phosphorus intake | 0% |
| Calcium intake | 7.3% |
| Sodium intake | 0% |
| Caffeine | 93.0% |
| Physical activity | 83.4% |
| Smoking status | 6.6% |
| CHF | 8.9% |
| CHD | 8.9% |
| Diabetes | 0% |
| Hypertension | 0.1% |
| Hyperlipidemia | 19.9% |
| Renal impairment | 14.9% |
| Malignancy | 8.9% |

Table S1. Missing data percentages for potential confounders

After excluding all participants with missing data, a total of 4,481 participants were included in the analysis. The analyses were repeated, yielding consistent results. The detailed results are as follows:

|  | **Model 1** |  | **Model 2** |  | **Model 3** |  |
| --- | --- | --- | --- | --- | --- | --- |
|  | HR (95% CI) | *p* | HR (95% CI) | *p* | HR (95% CI) | *p* |
| Continuous |  | 0.003 |  | 0.012 |  | 0.027 |
| Quartile1 | reference |  | reference |  | reference |  |
| Quartile2 | 0.55 (0.31, 0.97) | 0.038 | 0.60 (0.34, 0.94) | 0.048 | 0.61 (0.34, 0.97) | 0.045 |
| Quartile3 | 0.50 (0.25, 0.76) | 0.016 | 0.56 (0.39, 0.84) | 0.033 | 0.54 (0.39, 0.85) | 0.035 |
| Quartile4 | 0.24 (0.12, 0.46) | ＜0.001 | 0.27 (0.13, 0.57) | 0.001 | 0.29 (0.13, 0.64) | 0.002 |
| *p* for trend | ＜0.001 |  | 0.002 |  | 0.012 |  |

Table S2. Association between dietary phosphorus intake and cardiovascular mortality

Model 1: Adjusted for age, gender, race, and BMI.

Model 2: Additional adjustment was made for smoking status, hypertension, hyperlipidemia, chronic heart failure, coronary heart disease, diabetes, renal impairment, and malignancy.

Model 3: Further adjustment was made for total energy intake, saturated fat intake, sodium intake, calcium intake, serum calcium, and serum phosphorus.

Abbreviations: HR, hazard ratio; CI, confidence interval.


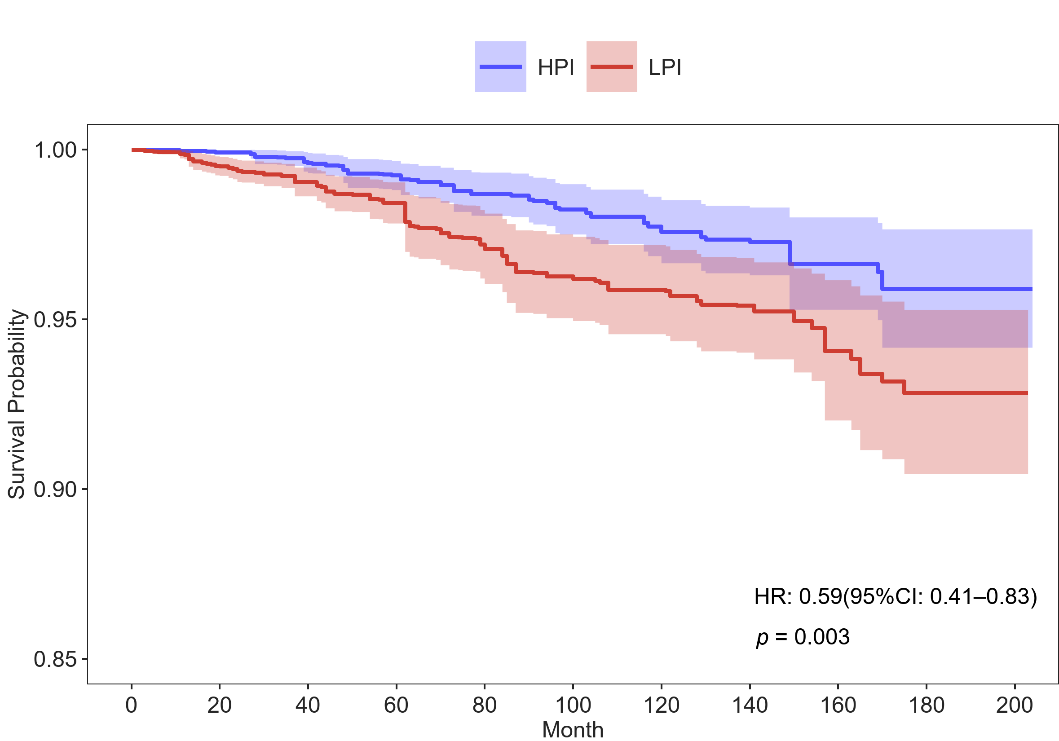


Figure S1. Kaplan-Meier survival curves for cardiovascular mortality in Model 3

Participants were divided into higher and lower phosphorus intake groups based on the median intake.

Abbreviations: HPI: higher phosphorus intake; LPI: lower phosphorus intake; HR, hazard ratio; CI, confidence interval.


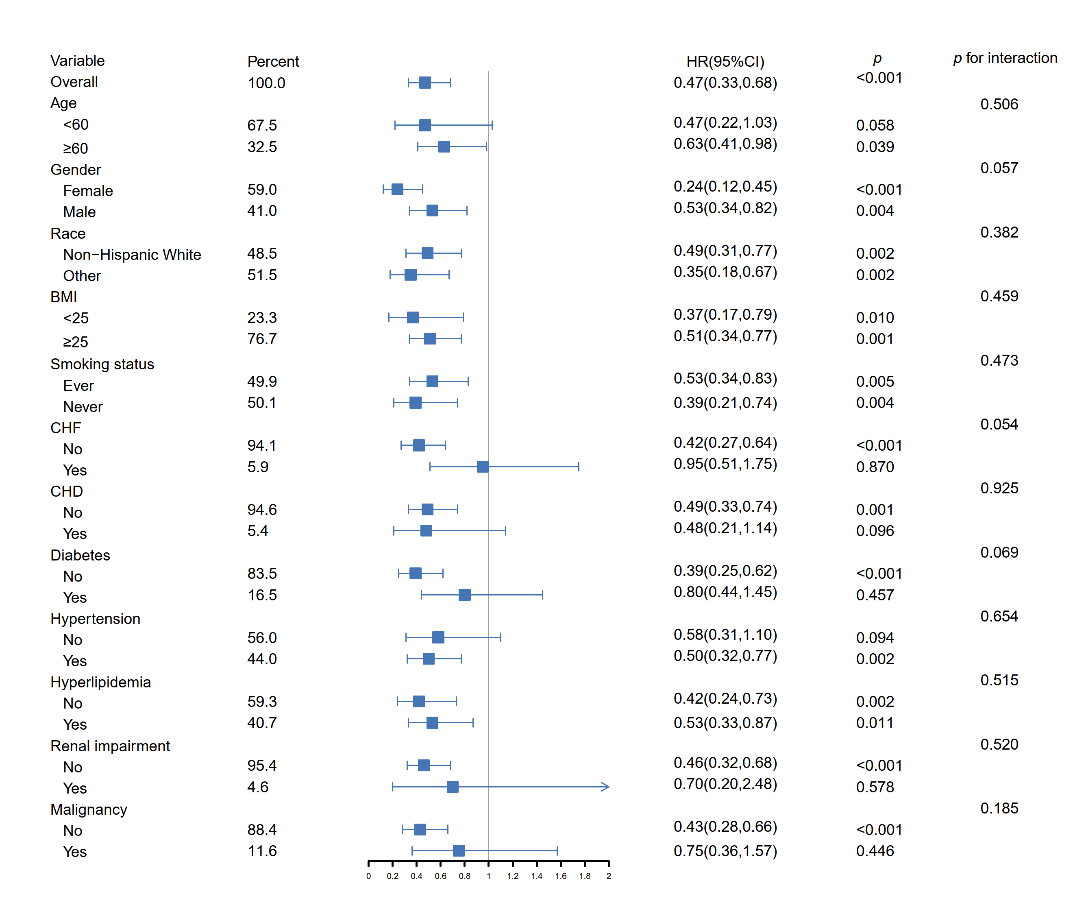


Figure S2. Subgroup analysis of the association between dietary phosphorus intake and cardiovascular mortality

Abbreviations: HR, hazard ratio; CI, confidence interval; BMI, body mass index; CHF, chronic heart failure; CHD, coronary heart disease.


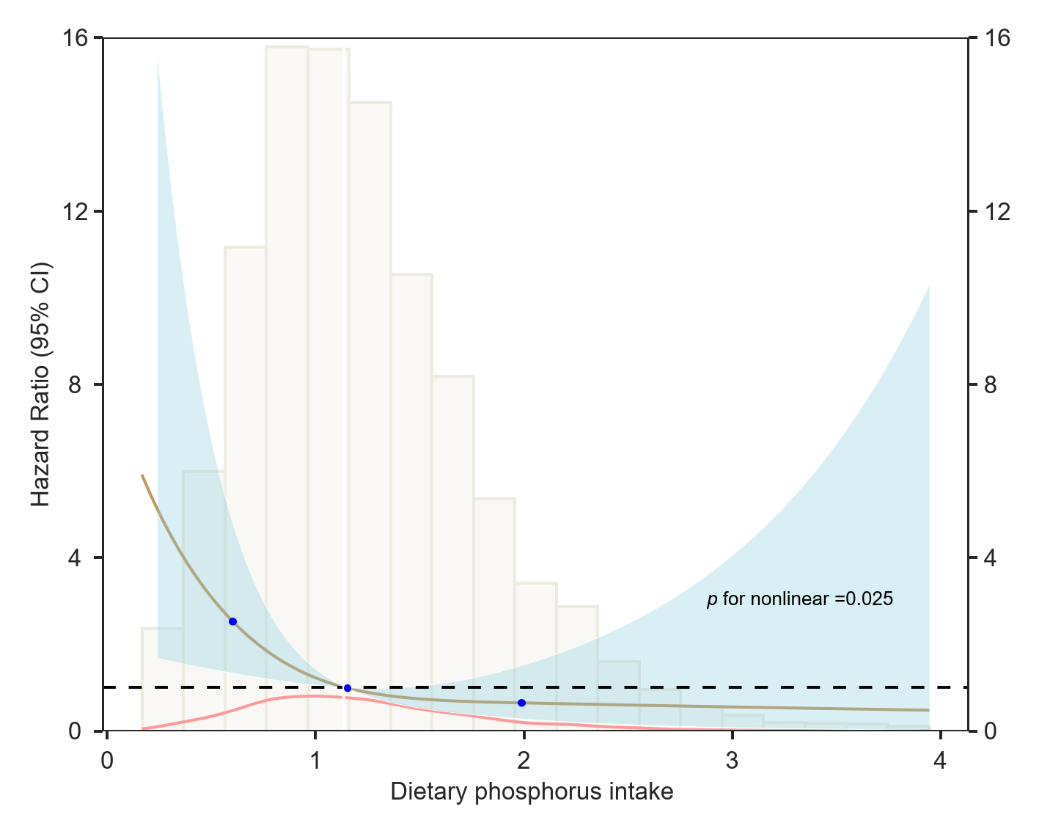


Figure S3. Restricted cubic spline of the relationship between dietary phosphorus intake and cardiovascular mortality risk in Model 3
